# Isopropyl 2-(5-iodo-7-methyl-3-methyl­sulfinyl-1-benzofuran-2-yl)acetate

**DOI:** 10.1107/S1600536808038671

**Published:** 2008-11-26

**Authors:** Hong Dae Choi, Pil Ja Seo, Byeng Wha Son, Uk Lee

**Affiliations:** a Department of Chemistry, Dongeui University, San 24 Kaya-dong Busanjin-gu, Busan 614-714, Republic of Korea; b Department of Chemistry, Pukyong National University, 599-1 Daeyeon 3-dong Nam-gu, Busan 608-737, Republic of Korea

## Abstract

In the title mol­ecule, C_15_H_17_IO_4_S, the O atom and the methyl group of the methyl­sulfinyl substituent lie on opposite sides of the plane of the benzofuran fragment. In the crystal structure, inter­molecular I⋯O [2.994 (3) Å] halogen bonding links the mol­ecules into centrosymmetric dimers, which are further packed into ribbons along the *c* axis by inter­molecular sulfin­yl–sulfinyl inter­actions [S⋯O 3.128 (3) Å].

## Related literature

For the crystal structures of similar isopropyl 2-(3-methyl­sulfinyl-1-benzofuran-2-yl)acetate derivatives, see Choi *et al.* (2008*a* ▶,*b* ▶). For a review of halogen bonding, see Politzer *et al.* (2007 ▶). For a review of carbon­yl–carbonyl inter­actions, see Allen *et al.* (1998 ▶).
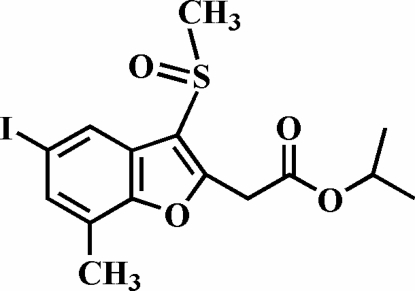


## Experimental

### 

#### Crystal data

C_15_H_17_IO_4_S
*M* *_r_* = 420.25
Monoclinic, 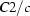

*a* = 17.615 (2) Å
*b* = 10.0905 (7) Å
*c* = 19.144 (1) Å
β = 99.177 (2)°
*V* = 3359.2 (5) Å^3^
*Z* = 8
Mo *K*α radiation
μ = 2.04 mm^−1^
*T* = 298 (2) K
0.40 × 0.30 × 0.20 mm

#### Data collection

Bruker SMART CCD diffractometer
Absorption correction: multi-scan (*SADABS*; Sheldrick, 1999 ▶) *T*_min_ = 0.480, *T*_max_ = 0.667
6667 measured reflections
2897 independent reflections
2172 reflections with *I* > 2σ(*I*)
*R*_int_ = 0.030

#### Refinement

*R*[*F* ^2^ > 2σ(*F* ^2^)] = 0.033
*wR*(*F* ^2^) = 0.064
*S* = 1.24
2897 reflections
192 parameters
H-atom parameters constrained
Δρ_max_ = 0.48 e Å^−3^
Δρ_min_ = −0.37 e Å^−3^

### 

Data collection: *SMART* (Bruker, 2001 ▶); cell refinement: *SAINT* (Bruker, 2001 ▶); data reduction: *SAINT*; program(s) used to solve structure: *SHELXS97* (Sheldrick, 2008 ▶); program(s) used to refine structure: *SHELXL97* (Sheldrick, 2008 ▶); molecular graphics: *ORTEP-3* (Farrugia, 1997 ▶) and *DIAMOND* (Brandenburg, 1998 ▶); software used to prepare material for publication: *SHELXL97*.

## Supplementary Material

Crystal structure: contains datablocks global, I. DOI: 10.1107/S1600536808038671/cv2478sup1.cif

Structure factors: contains datablocks I. DOI: 10.1107/S1600536808038671/cv2478Isup2.hkl

Additional supplementary materials:  crystallographic information; 3D view; checkCIF report

## Figures and Tables

**Table 1:** Selected interatomic distances (Å)

I⋯O4^i^	2.994 (3)
S⋯O4^ii^	3.128 (3)

Symmetry codes: (i) 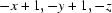; (ii) 
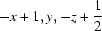
.

**Fig. 1.**
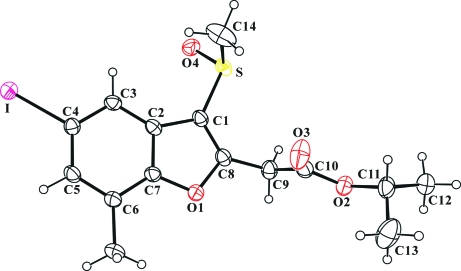
The molecular structure of the title compound, showing displacement ellipsoids drawn at the 30% probability level.

**Fig. 2.**
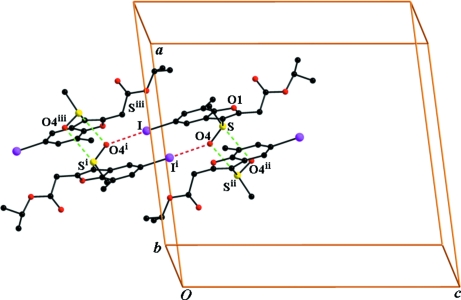
A portion of the crystal packing showing the I···O halogen bonding and S···O interactions by dotted lines [symmetry codes: (i) -x+1, -y+1, -z; (ii) -x+1, y, -z+1/2; (iii) x, -y+1, z-1/2].

## supplementary crystallographic information

### Comment

This work is related to our previous communications on the synthesis and structure of isopropyl 2-(3-methylsulfinyl-1-benzofuran-2-yl)acetate analogues, *viz*. isopropyl 2-(5-methyl-3-methylsulfinyl-1-benzofuran-2-yl)acetate (Choi *et al.*, 2008a) and isopropyl 2-(5-bromo-3-methylsulfinyl-1-benzofuran-2-yl)acetate (Choi *et al.*, 2008b). Here we report the crystal structure of the title compound, isopropyl 2-(5-iodo-7-methyl-3-methylsulfinyl-1-benzofuran-2-yl)acetate (Fig. 1).

The benzofuran unit is essentially planar, with a mean deviation of 0.030 (3) Å from the least-squares plane defined by the nine constituent atoms. The molecular packing (Fig. 2) is stabilized by intermolecular I···O halogen bonding (Politzer *et al.*,  2007) of 2.994 (3) Å and a nearly linear C—I···O angle of 168.51 (9)°, which link the molecules into centrosymmetric dimers (Table 1). These dimers are further packed into ribbons along the *c* axis by sulfinyl–sulfinyl interactions (Table 1) interpreted as simliar to a type–II carbonyl–carbonyl interaction (Allen *et al.*, 1998).

### Experimental

77% 3-Chloroperoxybenzoic acid (123 mg, 0.55 mmol) was added in small portions to a stirred solution of isopropyl 2-(5-iodo-7-methyl-3-methylsulfanyl-1-benzofuran-2-yl)acetate (202 mg, 0.5 mmol) in dichloromethane (30 ml) at 273 K. After being stirred for 3 h at room temperature, the mixture was washed with saturated sodium bicarbonate solution and the organic layer was separated, dried over magnesium sulfate, filtered and concentrated in vacuum. The residue was purified by column chromatography (ethyl acetate) to afford the title compound as a colorless solid [yield 81%, m.p. 396-397 K; *R*_f_ = 0.74 (ethyl acetate)]. Single crystals suitable for X-ray diffraction were prepared by evaporation of a solution of the title compound in acetone at room temperature. Spectroscopic analysis: ^1^H NMR (CDCl_3_, 400 MHz) δ 1.27 (d, J = 6.24 Hz, 6H), 2.46 (s, 3H), 3.06 (s, 3H), 4.00 (s, 2H), 5.03-5.09 (m, 1H), 7.49 (s, 1H), 8.10 (s, 1H); EI-MS 420 [M^+^].

### Refinement

All H atoms were geometrically positioned and refined using a riding model, with C—H = 0.93 Å for the aryl, 0.97 Å for the methylene, 0.98 Å for the methine, and 0.96 Å for the methyl H atoms. Uiso(H) = 1.2Ueq(C) for the aryl, methine and methylene H atoms, and 1.5Ueq(C) for methyl H atoms.

### Crystal data

**Table:**

C_15_H_17_IO_4_S	*F*_000_ = 1664
*M**_r_* = 420.25	*D*_x_ = 1.662 Mg m^−^^3^
Monoclinic, *C*2/*c*	Melting point = 420–421 K
Hall symbol: -C 2yc	Mo *K*α radiation λ = 0.71073 Å
*a* = 17.615 (2) Å	Cell parameters from 5394 reflections
*b* = 10.0905 (7) Å	θ = 2.2–28.1º
*c* = 19.144 (1) Å	µ = 2.04 mm^−^^1^
β = 99.177 (2)º	*T* = 298 (2) K
*V* = 3359.2 (5) Å^3^	Block, colourless
*Z* = 8	0.40 × 0.30 × 0.20 mm

### Data collection

**Table:**

Bruker SMART CCD diffractometer	2897 independent reflections
Radiation source: fine-focus sealed tube	2172 reflections with *I* > 2σ(*I*)
Monochromator: graphite	*R*_int_ = 0.030
Detector resolution: 10.0 pixels mm^-1^	θ_max_ = 26.0º
*T* = 298(2) K	θ_min_ = 2.5º
φ and ω scans	*h* = −12→21
Absorption correction: multi-scan(SADABS; Sheldrick, 1999)	*k* = −12→12
*T*_min_ = 0.480, *T*_max_ = 0.667	*l* = −23→23
6667 measured reflections	

### Refinement

**Table:**

Refinement on *F*^2^	Secondary atom site location: difference Fourier map
Least-squares matrix: full	Hydrogen site location: difference Fourier map
*R*[*F*^2^ > 2σ(*F*^2^)] = 0.033	H-atom parameters constrained
*wR*(*F*^2^) = 0.064	*w* = 1/[σ^2^(*F*_o_^2^)]
*S* = 1.24	(Δ/σ)_max_ < 0.001
2897 reflections	Δρ_max_ = 0.49 e Å^−^^3^
192 parameters	Δρ_min_ = −0.37 e Å^−^^3^
Primary atom site location: structure-invariant direct methods	Extinction correction: none

### Special details

**Table:**

Geometry. All esds (except the esd in the dihedral angle between two l.s. planes) are estimated using the full covariance matrix. The cell esds are taken into account individually in the estimation of esds in distances, angles and torsion angles; correlations between esds in cell parameters are only used when they are defined by crystal symmetry. An approximate (isotropic) treatment of cell esds is used for estimating esds involving l.s. planes.
Refinement. Refinement of F^2^ against ALL reflections. The weighted R-factor wR and goodness of fit S are based on F^2^, conventional R-factors R are based on F, with F set to zero for negative F^2^. The threshold expression of F^2^ > 2sigma(F^2^) is used only for calculating R-factors(gt) etc. and is not relevant to the choice of reflections for refinement. R-factors based on F^2^ are statistically about twice as large as those based on F, and R- factors based on ALL data will be even larger.

### Fractional atomic coordinates and isotropic or equivalent isotropic displacement parameters (Å^2^)

**Table:**

	*x*	*y*	*z*	*U*_iso_*/*U*_eq_	
I	0.513353 (18)	0.743648 (18)	−0.024404 (13)	0.04774 (10)	
S	0.60070 (6)	0.34161 (6)	0.23510 (5)	0.0450 (2)	
O1	0.61238 (16)	0.71392 (18)	0.29955 (13)	0.0445 (7)	
O2	0.71791 (17)	0.4874 (2)	0.48324 (13)	0.0503 (7)	
O3	0.7643 (2)	0.5249 (3)	0.38247 (15)	0.0783 (10)	
O4	0.53494 (17)	0.3086 (2)	0.17899 (13)	0.0554 (7)	
C1	0.6014 (2)	0.5163 (3)	0.24557 (19)	0.0408 (9)	
C2	0.5868 (2)	0.6160 (2)	0.19074 (19)	0.0382 (9)	
C3	0.5656 (2)	0.6170 (3)	0.11784 (18)	0.0389 (9)	
H3	0.5604	0.5386	0.0920	0.047*	
C4	0.5524 (2)	0.7395 (2)	0.0848 (2)	0.0394 (8)	
C5	0.5640 (2)	0.8584 (3)	0.12417 (19)	0.0430 (9)	
H5	0.5567	0.9388	0.1003	0.052*	
C6	0.5853 (2)	0.8597 (3)	0.19600 (19)	0.0414 (9)	
C7	0.5950 (2)	0.7356 (2)	0.2277 (2)	0.0385 (8)	
C8	0.6142 (2)	0.5781 (3)	0.30852 (19)	0.0409 (9)	
C9	0.6276 (2)	0.5310 (3)	0.3828 (2)	0.0470 (10)	
H9A	0.6021	0.4463	0.3851	0.056*	
H9B	0.6042	0.5932	0.4116	0.056*	
C10	0.7106 (3)	0.5155 (3)	0.4138 (2)	0.0452 (10)	
C11	0.7939 (3)	0.4653 (4)	0.5226 (2)	0.0575 (11)	
H11	0.8262	0.4217	0.4922	0.069*	
C12	0.7830 (3)	0.3742 (4)	0.5829 (2)	0.0690 (13)	
H12A	0.7593	0.2933	0.5642	0.083*	
H12B	0.7507	0.4166	0.6121	0.083*	
H12C	0.8321	0.3548	0.6106	0.083*	
C13	0.8288 (3)	0.5958 (5)	0.5478 (3)	0.0918 (17)	
H13A	0.7971	0.6382	0.5775	0.110*	
H13B	0.8325	0.6513	0.5077	0.110*	
H13C	0.8792	0.5813	0.5741	0.110*	
C14	0.5999 (3)	0.9856 (3)	0.2391 (2)	0.0615 (12)	
H14A	0.5901	0.9698	0.2863	0.092*	
H14B	0.5664	1.0544	0.2176	0.092*	
H14C	0.6525	1.0125	0.2407	0.092*	
C15	0.6855 (3)	0.3293 (4)	0.1950 (3)	0.0887 (19)	
H15A	0.6947	0.2381	0.1848	0.133*	
H15B	0.7286	0.3636	0.2269	0.133*	
H15C	0.6787	0.3796	0.1519	0.133*	

### Atomic displacement parameters (Å^2^)

**Table:**

	*U*^11^	*U*^22^	*U*^33^	*U*^12^	*U*^13^	*U*^23^
I	0.0565 (2)	0.04572 (12)	0.04033 (15)	−0.00107 (11)	0.00570 (13)	−0.00037 (10)
S	0.0481 (7)	0.0287 (3)	0.0571 (6)	−0.0014 (4)	0.0050 (5)	0.0060 (3)
O1	0.056 (2)	0.0353 (10)	0.0405 (15)	−0.0035 (10)	0.0028 (14)	0.0011 (9)
O2	0.0467 (19)	0.0623 (14)	0.0407 (16)	0.0041 (13)	0.0029 (14)	0.0026 (12)
O3	0.050 (2)	0.133 (2)	0.054 (2)	0.0157 (18)	0.0137 (18)	0.0208 (17)
O4	0.073 (2)	0.0443 (11)	0.0475 (17)	−0.0087 (12)	0.0045 (16)	−0.0040 (11)
C1	0.042 (2)	0.0309 (13)	0.048 (2)	−0.0029 (14)	0.0013 (18)	0.0044 (13)
C2	0.036 (2)	0.0290 (13)	0.049 (2)	−0.0007 (13)	0.0047 (19)	0.0011 (13)
C3	0.041 (2)	0.0303 (13)	0.044 (2)	−0.0048 (13)	0.0030 (19)	−0.0053 (13)
C4	0.038 (2)	0.0395 (15)	0.040 (2)	−0.0008 (14)	0.0057 (18)	0.0016 (13)
C5	0.049 (3)	0.0282 (13)	0.051 (2)	−0.0017 (14)	0.007 (2)	0.0047 (14)
C6	0.049 (3)	0.0318 (14)	0.042 (2)	−0.0027 (14)	0.0035 (19)	−0.0004 (13)
C7	0.040 (2)	0.0319 (14)	0.041 (2)	−0.0033 (13)	0.0007 (18)	−0.0019 (13)
C8	0.038 (2)	0.0351 (14)	0.048 (2)	−0.0014 (14)	0.0022 (19)	0.0053 (14)
C9	0.046 (3)	0.0473 (16)	0.047 (2)	−0.0063 (17)	0.004 (2)	0.0062 (15)
C10	0.047 (3)	0.0417 (15)	0.047 (2)	0.0019 (16)	0.008 (2)	0.0035 (15)
C11	0.047 (3)	0.078 (2)	0.045 (3)	0.019 (2)	−0.002 (2)	0.0008 (19)
C12	0.084 (4)	0.068 (2)	0.051 (3)	0.014 (2)	−0.002 (3)	0.0033 (19)
C13	0.077 (4)	0.117 (4)	0.076 (4)	−0.037 (3)	−0.004 (3)	0.007 (3)
C14	0.086 (4)	0.0306 (14)	0.064 (3)	−0.0021 (17)	−0.002 (2)	−0.0057 (15)
C15	0.073 (4)	0.052 (2)	0.151 (6)	0.006 (2)	0.049 (4)	−0.001 (2)

### Geometric parameters (Å, °)

**Table:**

I—C4	2.095 (4)	C6—C14	1.514 (4)
I—O4^i^	2.994 (3)	C8—C9	1.483 (5)
S—O4	1.486 (3)	C9—C10	1.496 (5)
S—O4^ii^	3.128 (3)	C9—H9A	0.9700
S—C1	1.773 (3)	C9—H9B	0.9700
S—C15	1.789 (4)	C11—C13	1.500 (6)
O1—C7	1.378 (4)	C11—C12	1.511 (5)
O1—C8	1.381 (3)	C11—H11	0.9800
O2—C10	1.346 (4)	C12—H12A	0.9600
O2—C11	1.445 (6)	C12—H12B	0.9600
O3—C10	1.201 (4)	C12—H12C	0.9600
C1—C8	1.344 (5)	C13—H13A	0.9600
C1—C2	1.447 (5)	C13—H13B	0.9600
C2—C3	1.386 (5)	C13—H13C	0.9600
C2—C7	1.395 (4)	C14—H14A	0.9600
C3—C4	1.392 (4)	C14—H14B	0.9600
C3—H3	0.9300	C14—H14C	0.9600
C4—C5	1.414 (4)	C15—H15A	0.9600
C5—C6	1.367 (5)	C15—H15B	0.9600
C5—H5	0.9300	C15—H15C	0.9600
C6—C7	1.390 (4)		
			
I···O4^i^	2.994 (3)	S···O4^ii^	3.128 (3)
			
O4—S—C1	107.19 (17)	H9A—C9—H9B	107.6
C4—I—O4^i^	168.51 (9)	O3—C10—O2	123.5 (4)
O4—S—C15	106.4 (2)	O3—C10—C9	126.3 (4)
C1—S—C15	97.22 (16)	O2—C10—C9	110.3 (3)
C7—O1—C8	106.2 (2)	O2—C11—C13	109.3 (3)
C10—O2—C11	118.8 (3)	O2—C11—C12	105.8 (4)
C8—C1—C2	108.2 (3)	C13—C11—C12	112.6 (4)
C8—C1—S	124.1 (3)	O2—C11—H11	109.7
C2—C1—S	127.7 (3)	C13—C11—H11	109.7
C3—C2—C7	119.6 (3)	C12—C11—H11	109.7
C3—C2—C1	136.4 (3)	C11—C12—H12A	109.5
C7—C2—C1	104.0 (3)	C11—C12—H12B	109.5
C2—C3—C4	117.6 (3)	H12A—C12—H12B	109.5
C2—C3—H3	121.2	C11—C12—H12C	109.5
C4—C3—H3	121.2	H12A—C12—H12C	109.5
C3—C4—C5	120.8 (4)	H12B—C12—H12C	109.5
C3—C4—I	118.4 (2)	C11—C13—H13A	109.5
C5—C4—I	120.7 (2)	C11—C13—H13B	109.5
C6—C5—C4	122.5 (3)	H13A—C13—H13B	109.5
C6—C5—H5	118.7	C11—C13—H13C	109.5
C4—C5—H5	118.7	H13A—C13—H13C	109.5
C5—C6—C7	115.2 (3)	H13B—C13—H13C	109.5
C5—C6—C14	123.5 (3)	C6—C14—H14A	109.5
C7—C6—C14	121.3 (3)	C6—C14—H14B	109.5
O1—C7—C6	124.8 (3)	H14A—C14—H14B	109.5
O1—C7—C2	110.9 (2)	C6—C14—H14C	109.5
C6—C7—C2	124.2 (4)	H14A—C14—H14C	109.5
C1—C8—O1	110.6 (3)	H14B—C14—H14C	109.5
C1—C8—C9	133.6 (3)	S—C15—H15A	109.5
O1—C8—C9	115.8 (3)	S—C15—H15B	109.5
C8—C9—C10	114.2 (3)	H15A—C15—H15B	109.5
C8—C9—H9A	108.7	S—C15—H15C	109.5
C10—C9—H9A	108.7	H15A—C15—H15C	109.5
C8—C9—H9B	108.7	H15B—C15—H15C	109.5
C10—C9—H9B	108.7		
			
O4—S—C1—C8	137.8 (3)	C5—C6—C7—C2	2.3 (6)
C15—S—C1—C8	−112.5 (4)	C14—C6—C7—C2	−176.2 (4)
O4—S—C1—C2	−40.3 (4)	C3—C2—C7—O1	176.9 (3)
C15—S—C1—C2	69.4 (4)	C1—C2—C7—O1	−0.6 (4)
C8—C1—C2—C3	−175.0 (4)	C3—C2—C7—C6	−1.9 (6)
S—C1—C2—C3	3.4 (7)	C1—C2—C7—C6	−179.4 (4)
C8—C1—C2—C7	1.9 (4)	C2—C1—C8—O1	−2.5 (4)
S—C1—C2—C7	−179.8 (3)	S—C1—C8—O1	179.1 (3)
C7—C2—C3—C4	−0.8 (5)	C2—C1—C8—C9	175.8 (4)
C1—C2—C3—C4	175.7 (4)	S—C1—C8—C9	−2.6 (6)
C2—C3—C4—C5	3.0 (5)	C7—O1—C8—C1	2.1 (4)
C2—C3—C4—I	−176.1 (2)	C7—O1—C8—C9	−176.6 (3)
C3—C4—C5—C6	−2.7 (6)	C1—C8—C9—C10	93.3 (5)
I—C4—C5—C6	176.4 (3)	O1—C8—C9—C10	−88.4 (4)
C4—C5—C6—C7	0.1 (5)	C11—O2—C10—O3	−0.9 (5)
C4—C5—C6—C14	178.5 (4)	C11—O2—C10—C9	178.4 (3)
C8—O1—C7—C6	178.0 (4)	C8—C9—C10—O3	−7.7 (5)
C8—O1—C7—C2	−0.8 (4)	C8—C9—C10—O2	172.9 (2)
C5—C6—C7—O1	−176.4 (3)	C10—O2—C11—C13	86.0 (4)
C14—C6—C7—O1	5.1 (6)	C10—O2—C11—C12	−152.5 (3)

Symmetry codes: (i) −*x*+1, −*y*+1, −*z*; (ii) −*x*+1, *y*, −*z*+1/2.
